# Anatomical background of ovine kidney for use as animal model: analysis of arterial segmentation, proportional volume of each segment and arterial injury after cranial pole partial nephrectomy

**DOI:** 10.1590/S1677-5538.IBJU.2019.0842

**Published:** 2020-09-02

**Authors:** Daniel H. Zidde, Francisco J. B. Sampaio, Paulo de Souza, Diogo B. de Souza, Marco A. Pereira-Sampaio

**Affiliations:** 1 Unidade de Pesquisa Urogenital Universidade Estadual do Rio de Janeiro Rio de JaneiroRJ Brasil Unidade de Pesquisa Urogenital, Universidade Estadual do Rio de Janeiro - Uerj, Rio de Janeiro, RJ, Brasil;; 2 Laboratório de Anatomia Animal Universidade Federal do Pampa UruguaianaRS Brasil Laboratório de Anatomia Animal, Universidade Federal do Pampa, Uruguaiana, RS, Brasil;; 3 Departamento de Morfologia Universidade Federal Fluminense NiteróiRJ Brasil Departamento de Morfologia, Universidade Federal Fluminense - UFF, Niterói, RJ, Brasil

**Keywords:** Anatomy, Models, Animal, Kidney

## Abstract

**Objective:**

To study the arterial segments of ovine kidney, present a proportional volume analysis of each kidney arterial segment, and analyze arterial injuries caused by simulated partial nephrectomy of cranial pole.

**Materials and Methods:**

Forty-eight ovine kidneys injected with polyester resin into the renal arteries and collecting system were used in this study. Eighteen kidneys were used to study the arterial segments and the proportional volume of each renal segment. Other 30 kidneys were submitted to superior pole resection at a distance of 1.0cm, 0.5cm, or exactly at the cranial hilar edge, just before the resin hardening. These endocasts were used to evaluate the arterial injuries caused by these different resection planes.

**Results:**

Ovine renal artery divided into two (ventral and dorsal) or three segmental arteries. Dorsal segment presented higher proportional volume than ventral segment. For kidneys with three segments, the third segment was on the caudal region (caudo-ventral or caudo-dorsal segment) and presented the lowest proportional volume. None of the resected kidneys (at 1.0, 0.5 or at the cranial hilar edge) presented injury of arterial branches that irrigate non-resected region.

**Conclusion:**

The segmental distribution of renal artery, the proportional volume of each segment and arterial injuries after cranial pole resection in ovine kidneys are different from what is observed in human kidneys. Meanwhile, ovine kidneys show a primary segmental division on anterior and posterior, as in humans, but different from swine. These anatomical characteristics should be considered when using ovine as animal models for renal experimental and/or training procedures.

## INTRODUCTION

The pig is the animal model more traditionally used when performing research and training of renal procedures ( 1 , 2 ). This fact is based on some anatomical resemblances with human kidney, especially regarding its size and collecting system anatomy ( 3 ). However, there are some important anatomical differences between the kidneys of humans and swine, which advise against the use of pigs as animal model for some studies.

The renal artery division into segmental arteries ( 4 ) and the relationship between the posterior segmental artery and cranial infundibulum in pigs ( 5 ) are distinct from what is observed in human kidneys ( 6 ). Add to this, it is already demonstrated that swine kidney heals very differently from human’s after partial nephrectomies ( 7 ) and it is not an adequate model for studying hemostatic methods for kidney procedures ( 8 ). These differences led to a searching for other animal models, which could substitute pigs for renal procedures training and research purposes.

Since it was showed that the ovine kidney heals after partial nephrectomies in a more similar way of what is observed in humans, sheep became to be considered as animal models for renal studies ( 9 ). The intrarenal anatomy ( 10 ) and histological aspects of ovine kidney ( 11 ) were described, depicting similarities and differences in comparison to the human kidney. However, deeper investigation was not performed yet.

The knowledge on the anatomical trajectory of retropielic artery of the human kidney was important to understand and avoid the occurrence of devascularized areas of remaining renal parenchyma after upper pole partial nephrectomies. This artery commonly directs to the upper pole (on the posterior region), but turns down to irrigate the mesorenal region and inferior pole ( 12 ). Therefore, once sectioned for an upper pole tumor resection, non-resected areas of the mesorenal region and lower pole can be left without arterial supply. This is an important anatomical aspect that should be taken into consideration when choosing an animal model for studying or training cranial pole nephrectomies. It is still unknown if resections of ovine kidneys cranial pole may have similar vascular consequences as observed in human kidneys.

Further, it is well detailed the proportions among the renal segments both in human ( 13 ) and swine ( 4 ), but not in ovine kidneys. This information is important to correlate functional losses with resected segments during partial nephrectomies, both in cranial, caudal pole, and mesorenal region. This information can be also important for deciding if ovine kidney is suitable as a model in nephrolithiasis and other procedures requiring renal parenchyma invasion.

The hypothesis of this study is that the ovine kidney may have anatomical similarities to human kidney that can be advantages to be explored for experimental and/or training purposes. Thus, the objectives of the present study were: to study the arterial segments of ovine kidney; to present a proportional volume analysis of each kidney arterial segment; and to analyze the vascular lesions due to simulated partial nephrectomy of the superior pole. Further, the results of this study were compared to human kidney analyses (already performed by our research group), depicting similarities and differences that may influence the choice of ovine as animal model for research and training on kidney procedures.

## MATERIALS AND METHODS

Forty-eight ovine kidneys were obtained from young adult Polwarth rams, weighting 43.2-48.5Kg slaughtered for commercial purposes. The kidneys were collected en bloc (with abdominal aorta, caudal vena cava and ureters) immediately after death, rinsed and frozen until utilization. Kidneys presenting any macroscopic alterations or lesions, either pathological or caused during slaughter, were discarded. The study was conducted in accordance to national and international laws for animal utilizations on research and was formally approved by the institutional ethics committee for animal experimentation (protocol no. 001/2018).

The analyses of the arterial segments of the ovine kidney and proportional volume of each segment were performed in 18 kidneys. The renal pedicle of these organs was dissected and extra-hilar branches of renal artery were identified and isolated. Then, each segmental artery was cannulated separately. Each artery was injected with polyester resin pigmented with different colors for each segment and 2.5% of catalyst (methyl ethyl peroxide) ( 14 ). The resin was manually injected under pressure with plastic disposable syringes, until each arterial segment was completely fulfilled.

**Figure 1:**
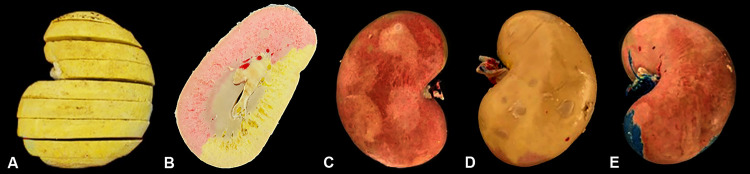
Ovine kidney prepared for proportional volume analysis of each segment. At image A, after resin injection of different colors into each segmental artery, the kidney was sectioned in 0.5-thick slices. At image B it is noted the surface of one transverse section, with the region irrigated by ventral (red) segmental artery and (yellow) segmental artery. Ventral (C) and dorsal (D) faces of two-segment ovine kidney, and ventral face of three-segment ovine kidney (E) after resin injection of different colors into each segmental artery.

After the hardening of the resin, the kidneys were dissected from its appendices, immersed in 4% formaldehyde for 48 hours and then frozen. The frozen kidneys were transversally sliced with a table-band-saw, obtaining 0.5cm- thick sequential sections ( Figure-1 ). Each section was photographed and Cavalieri’s principle was used for measuring the proportional volume of each renal segment (stained with different pigments). Briefly, in each photography the demarked areas of each color, as well as the total slice surface, were measured with the “free-hand-selection” tool of ImageJ software (Version 1.37, NIH). The sum of each segment area (of all sections) was divided by the sum of the entire slice surface (of all sections) for obtaining the proportional volume (expressed in percentage) of each renal segment ( 15 , 16 ). The proportional volume of renal segments from right and left kidneys (which has the same number of segments) was compared.

Other 30 kidneys were used for analysis of vascular lesions due to simulated partial nephrectomy of the superior pole ( 17 , 18 ). These organs were divided into three groups: Group 1 - kidneys sectioned exactly at the cranial hilar edge (n=10); Group 2 - kidneys sectioned at 0.5cm from the hilar edge (n=9); and Group 3 kidneys sectioned at 1.0cm from the hilar edge (n=11).

For these kidneys, after dissection of renal pedicle, the renal artery and ureter were cannulated and injected with the same resin compound described for the previous experiment. A yellow pigment was standardized for the resin injected into the collecting system, and a red pigment for the compound injected into the renal artery.

After injection, the kidneys were dissected from its appendices. Before the total hardening of the resin (about 15 minutes after the addition of the catalyst), the cranial pole was transversely (guillotine) sliced. The site of the section was determined according to the above-mentioned group division. After total resin hardening the renal parenchyma was digested by immersion in 50% concentrated commercial hydrochloric acid. This allows complete digestion of all tissues, leaving a tri-dimension renal endocast of the arterial and collecting systems ( 18 ). These endocasts were used to study the arterial branches (and collecting system) sectioned in each group. Special attention was given to identify non-resected areas that would become devascularized.

Descriptive statistical analysis was performed for all data. Data is presented as mean, maximum and minimum values, and coefficient of variation. Chi-square test was used to compare the proportional volume of right and left kidneys. Results were considered significant when p <0.05. All analyses were performed using GraphPad Prism software (version 5.0, San Diego, CA, USA).

## RESULTS

Regarding the arterial segments of the 18 studied kidneys, 88.8% (16 kidneys) presented a division of renal artery into two segmental arteries. One destinated to the ventral (anterior) region, and other for the dorsal (posterior) region. In 11.2% (two kidneys) there was three segmental arteries ( Figure-1 ). In one of these, besides the dorsal and ventral segmental arteries, a third one was observed irrigating the dorsal region of caudal pole (caudo-dorsal segment); while in the other kidney with three segments there was a dorsal segment, a ventral segment and a caudo-ventral segment, irrigating the ventral region of caudal pole.

The analyses of proportional volume of each kidney arterial segment showed that in kidneys with two segments, the ventral segment corresponds to 48.85% and dorsal segment to 51.15% of renal volume (mean). For the kidneys with three segments, ventral segment represents 39.60% of renal volume, dorsal segment 42.77% and caudo-dorsal or caudo-ventral comprises 17.62% (mean). When two and three-segmented kidneys are analyzed together, it is observed that ventral segment comprises 47.82% of renal volume, dorsal segment corresponds to 50.22% and caudo-ventral or caudo-dorsal 17.62% (mean).

Both kidneys with three segments were left-sided, nevertheless, the number of three-segmented kidneys (2 organs) is too few to perform statistical analysis. When the proportional volume of ventral and dorsal segments of right and left kidneys (with two segments) were compared, no statistical difference was observed (p=0.887). Table-1 presents the results of proportional volume of each renal segment analyses with the descriptive statistics data.

**Table 1:** Proportional and absolute volumes of renal segments and renal volume of ovine kidneys.

Kidneys with two arterial segments (n=16)				
	Mean	Maximum	Minimum	C.V.
Ventral segment P.V. (%)	48.85	66.27	41.66	12.38
Dorsal segment P.V. (%)	51.15	58.33	33.72	11.84
**Kidneys with three arterial segments (n=2)**				
	**Mean**	**Maximum**	**Minimum**	**C.V.**
Ventral segment P.V. (%)	39.60	42.68	36.52	11.01
Dorsal segment P.V. (%)	42.77	47.05	38.49	14.15
Caudal segment* P.V. (%)	17.62	18.82	16.42	9.61
**All kidneys (n=18)**				
	**Mean**	**Maximum**	**Minimum**	**C.V.**
Ventral segment P.V. (%)	47.82	66.27	36.52	13.60
Dorsal segment P.V. (%)	50.22	58.33	33.72	12.87
Caudal segment* P.V. (%)	17.62	18.82	16.42	9.61

**P.V.** = Proportional Volume; **C.V.** = Coeficient of Variation; * = Caudal segment refers to caudo-ventral and caudo-dorsal segments.

**Figure 2:**
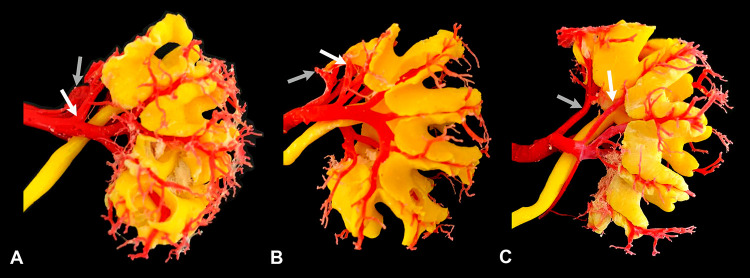
Dorsal aspect of ovine renal endocast, obtained from kidneys sectioned at the hilar edge (A), at 0.5cm (B), and at 1.0cm from the hilar edge (C). At images A and B it is noted that two cranial branches of dorsal (white arrow) and ventral segmental arteries (gray arrow) were sectioned, without prejudice to non-resected areas. At image C it is noted that most cranial branches of dorsal (white arrow) and ventral segmental arteries (gray arrow) are not injured when the kidney is sectioned at 1cm from the hilar edge.

Regarding the analysis of the vascular lesions due to simulated partial nephrectomy of the superior pole, none of the kidneys (either sectioned at 0.5 or 1.0cm or exactly at the hilar edge) presented arterial injuries in branches that irrigates mesorenal region or caudal pole ( Figure-2 ). In all organs, only the resected regions lost their vascularization.

## DISCUSSION

This study presents important and unpublished information about the ovine renal anatomy. Such information, besides serving as a basic study of anatomy for this species, gains more importance because it can be used for comparison with other species, especially with human kidneys. In this sense, this article points out advantages and disadvantages of using ovine as an animal model for study and training of renal surgeries, depending on the factor that should be considered.

All ovine kidneys had a single renal artery in all cases. The single renal artery is also found in pigs ( 5 ) and rabbits ( 19 ), but in dogs two or three renal arteries may be found for a single kidney ( 20 ). In humans, only 53.3% of the kidneys have a single renal artery, with two or three hilar and extra-hilar arteries for both poles in the other cases ( 21 ). This difference in the number of renal arteries should be considered when using ovine kidneys for training surgical procedures in which the number of renal arteries is an important factor, such as laparoscopic nephrectomy for kidney transplant donors, as the surgeon will not find multiple renal arteries, as in the human patient ( 21 ).

By evaluating the endocasts of the arteries and the collecting system after the simulated nephrectomy, we observed that only the interlobar arteries for the resected areas of the kidney were injured. Therefore, in none of the cases there was an arterial lesion leaving part of the remaining parenchyma without vascularization. This is an important aspect since in human kidneys a recurrent trajectory, of retropielic artery, is observed ( 17 ). Thus, for the purpose of partial nephrectomy training, it is desirable to have animal models that have vascular anatomy that provides lesions to the remaining parenchyma like pigs ( 18 ), and can simulate this type of complication as in humans.

In our previous study with porcine kidney, the arterial distribution of the dorsal and ventral mesorenal regions was compromised in 13.33% and 20% of the cases respectively when the kidney was sectioned in the hilum. When the organ was sectioned at 0.5cm cranial to the renal hilum, the dorsal mesorenal region was compromised in 6.25% of the cases. On the other hand, in nephrectomies performed at 1.0cm and 1.5cm cranially to the hilum, there was no lesion in the cranial division of the renal artery and the arterial supply to the midzone of the kidney was preserved as in humans ( 18 ). Thus, although the pig kidney does not have a retropielic artery, it remains the best animal model for partial upper pole nephrectomy when vascular injury is an important point for the study, and it is still the most used model in partial nephrectomies ( 22 - 24 ).

Studies on the proportional area of the arterial segments have shown that the main arterial segment in humans is the posterior ( 13 ), while in pigs the main arterial segment is the cranial ( 4 ). These studies have shown that the proportion of each renal segment is very distinct between these two species, and this is a negative factor for using swine in some renal studies.

By the other hand, in ovine kidneys the renal artery is divided into ventral and dorsal branches, more similar to that found in the kidneys of humans ( 6 ), dogs ( 20 ) and rabbits ( 19 ). Considering that ovine and its kidneys are closer to human in size and weight (compared to rabbits and dogs), this species may be considered the best model for renal studies in which segmental division is an important factor. This species may be particularly interesting for using in anatrophic nephrolithotomy studies. As it was observed, the division on anterior and posterior segments, leaves a (so-called) avascular plane (Brodel’s line) similarly to what is found in human kidney ( 25 ).

Currently, there is a tendency for reducing animals in experimentation and replacing animals for artificial models or digital simulators ( 26 , 27 ). Although this is an important trend, in many studies the use of animals is still necessary. In this way, the present study aims to refine the searching for animal models, avoiding the use of inadequate species.

The study of renal endocasts, obtained from resin injection and corrosion, is traditionally used for anatomical investigations ( 3 , 10 ). The major disadvantages of this method are that the whole organ needs to be extracted and corroded. This adds difficulties for obtaining samples (specially from humans), and do not allow any further tissue analysis (e.g. histological, molecular). Recently, renal endocasts are being obtaining by 3D printing and used for different purposes ( 28 - 30 ). The information of renal anatomy is commonly obtained by computer tomograms or magnetic resonance exams. The advantages of this method are that renal anatomy can be individually studied, with non-invasive methods, and it does not hamper other functional analysis or renal biopsy. Possible disadvantages are that it requires patient collaboration for performing the exam (especially if the exam is not needed for any diagnostic), the whole process of obtaining the endocast is more expensive, and the endocast resolution is limited by the exam resolution, slice thickness and 3D printer resolution. In the present study, the traditional method was chosen as it has a better resolution and it is easier and cheaper to obtain ovine kidneys than imaging exams of it.

All kidneys were obtained from the same breed of rams, and this may be a limitation of the study. However, there is no information indicating that different breeds may have important differences in intrarenal anatomy in ovine or any other species. There is also no evidence that females may have differences in intrarenal anatomy in ovine or other species. Thus, although the material has been limited to one race and one sex, at first this information may be extrapolated to other ovine breeds as well as females.

## CONCLUSIONS

This study presented results on the renal anatomy of rams and compared with the anatomy already described in other animal models and with the human kidney. All differences and similarities should be taken into consideration when choosing the best animal model according to the research or training purpose.

The ovine kidney differs from the human kidney as it has a single renal artery, a distinct segmental distribution and absence of arteries with a pathway that provides ischemic lesions to the remaining parenchyma after partial nephrectomy of the cranial pole. On the other hand, the ovine kidney is similar to the human kidney as it has a primary division of the renal artery in anterior and posterior segments, with similar proportional volume of these regions.
